# Antibiotic therapy for skin and soft tissue infections: a protocol for a systematic review and network meta-analysis

**DOI:** 10.1186/s13643-018-0804-8

**Published:** 2018-09-11

**Authors:** Jessica J. Bartoszko, Dominik Mertz, Lehana Thabane, Mark Loeb

**Affiliations:** 1 Department of Health Research Methods, Evidence and Impact, McMaster University, Hamilton, Ontario L8N 3Z5 Canada; 2 Department of Medicine, McMaster University, Hamilton, Ontario L8S 4K1 Canada; 3 Institute for Infectious Disease Research, McMaster University, Hamilton, Ontario L8S 4L8 Canada; 4 Department of Pathology and Molecular Medicine, McMaster University, Hamilton, Ontario L8N 3Z5 Canada; 5 Departments of Anesthesia and Pediatrics, McMaster University, Hamilton, Ontario L8S 4L8 Canada; 6 Biostatistics Unit, St. Joseph’s Healthcare Hamilton, Hamilton, Ontario L8N 4A6 Canada; 7 Michael DeGroote Centre for Learning and Discovery (MDCL) - 3208, 1280 Main St W, Hamilton, Ontario L8S 4K1 Canada

**Keywords:** Skin and soft tissue infection, Antibiotic therapy, Systematic review, Network meta-analysis, Protocol

## Abstract

**Background:**

Skin and soft tissue infections (SSTIs) in hospital and community settings impose a substantial socio-economic burden. Therapeutic uncertainty due to the availability of a wide range of antibiotics and the need for empirical treatment decisions complicate SSTI clinical management. Completion of numerous pairwise meta-analyses to account for this variability in antibiotics is impractical, and many head-to-head comparisons of potential interest are likely not available. In comparing multiple antibiotics simultaneously, this network meta-analysis aims to identify the antibiotic(s) with the greatest value in the treatment of SSTIs, in terms of patient-important outcomes such as efficacy and safety.

**Methods:**

We will conduct a systematic review to identify randomized controlled trials of persons with suspected or confirmed SSTI assigned to orally or parenterally administered antibiotic therapy that report results on at least one outcome of interest. We will search MEDLINE, EMBASE, and the Cochrane Central Register of Controlled Trials (CENTRAL), along with trial registries. Our primary outcome of interest is clinical success at the test-of-cure visit. Secondary outcomes may include (1) early clinical success (2–3 days after the therapy starts), (2) mortality, (3) adverse events, (4) treatment duration, and (5) length of hospital stay. Independent reviewers will complete screening of titles, abstracts, and full texts, data extraction, risk of bias assessment (using the Cochrane Risk of Bias tool), and evaluation of the certainty of evidence (using the GRADE approach) in duplicate. We will complete pairwise and network meta-analyses within the Bayesian framework when possible using a random effects model. We will stratify SSTIs by severity into uncomplicated and complicated SSTIs when possible. Subgroup analyses by age, infection type, comorbidity, and suspected or confirmed methicillin-resistant *Staphylococcus aureus* (MRSA)-associated infection are planned.

**Discussion:**

This study has several strengths compared to previous reviews: inclusion of a wider range of infection types, antibiotics, and outcomes; a comprehensive search strategy; a priori subgroup analyses; application of GRADE; and improved interpretability of findings through visual presentation of results. We hope our findings will inform future research, health care professionals, and policy makers resulting in improved evidence-based clinical management of SSTIs.

**Systematic review registration:**

PROSPERO CRD42018085607

**Electronic supplementary material:**

The online version of this article (10.1186/s13643-018-0804-8) contains supplementary material, which is available to authorized users.

## Background

Skin and soft tissue infections (SSTIs) can differ in clinical presentation, microbial etiology, and severity. They can range from uncomplicated cases, such as cellulitis lacking systemic signs of infection, to more severe cases, such as diabetic foot infection involving the soft tissue in a vascular compromised host [1–6]. SSTIs caused by Gram-positive bacteria, such as *Staphylococcus aureus* and beta hemolytic streptococci, are the most common [5]. The number of SSTIs in hospital and community settings is increasing, and the cost burden from SSTIs for the health care system is significant [7–9]. The incidence of *Staphylococcus aureus*-related SSTI hospitalizations in the United States (US) significantly increased from 2001 to 2009 (57 to 117 cases/100,000 people; *p* < 0.01) and was associated with an increase in national annual cost (from 3.36 to 4.22 billion dollars) [8]. The Canadian Nosocomial Infection Surveillance Program reported an increase in hospital admissions for SSTI due to methicillin-resistant *Staphylococcus aureus* (MRSA) from 24 to 37% between 1994 and 2007 [9]. A retrospective study from 2001 to 2005 in the US demonstrated an increase in SSTIs from 20 to 61 per 1000 outpatient visits. An increase in the proportion of outpatients’ bacterial cultures positive for MRSA was also observed (4 to 42%) [10]. Similar trends, associated with increasing incidence of SSTI due to community-acquired MRSA, have also been observed in Canada [11–15]. SSTIs are among the most common diagnoses in emergency rooms, following chest pain and asthma, and are the cause of approximately 7 to 10% of hospital admissions in North America [3, 4].

Antibiotic therapy and severity stratification decisions for SSTIs are often based on clinical impression, before confirmatory laboratory testing, involving bacterial identification and antibiotic susceptibility testing, can be completed. Empirical treatment may curb clinical resolution, but is often a necessary intervention to prevent severe outcomes, such as comorbidity and death [1, 10]. The multiplicity of antibiotic therapies available, and the introduction of novel antibiotic therapies, such as tedizolid and dalbavancin, which have not been extensively studied in active-comparator trials, may pose additional challenges in selecting appropriate treatment [1, 2]. With this therapeutic uncertainty, it is critical that treatment recommendations and clinical management of SSTIs are founded from the best available evidence.

The Infectious Disease Society of America (IDSA) published guidelines for the treatment of SSTIs in 2014. These recommendations were based on evidence from randomized controlled trials (RCTs) and well-conducted observational studies, which an expert panel reviewed and evaluated [2]. Such studies, especially RCTs, are crucial in generating evidence, but may lead to misguided treatment decisions when used independently of each other [16]. With the wide range of antibiotics available for the treatment of SSTIs, quantitative synthesis of all the available evidence is warranted. Completion of numerous pairwise meta-analyses to account for this variety in antibiotics is impractical, requiring considerable effort and time. But most importantly, many head-to-head comparisons of potential interest are not likely available.

In network meta-analyses (NMAs), treatment options are compared directly (based on within trial comparisons) and indirectly (antibiotics not previously studied in head-to-head comparisons) through a common comparator between studies. Although NMAs are methodologically advanced, their clinical utility surpasses that of traditional meta-analyses that are limited to head-to-head comparisons. In comparing multiple antibiotics simultaneously by NMA, the antibiotic(s) with the greatest value in the treatment of SSTIs, in terms of patient-important outcomes such as efficacy and safety, can be identified [17].

We are aware of four related NMAs that have been conducted in the last decade; however, they were limited in the antibiotics and infection types studied, generalizability and methodological rigor [18–21]. To the best of our knowledge, there has been no review as comprehensive as the one proposed. The aim of this study is to synthesize all available evidence based on direct and indirect comparisons of patient-important outcomes that include clinical efficacy, mortality, safety, treatment duration, and length of hospital stay to identify the antibiotic(s) with the greatest value in the treatment of SSTIs among diverse populations improving evidence clinical management of SSTIs is based on.

## Methods/design

This protocol was written according to the Preferred Reporting Items for Systematic Review and Meta-Analysis Protocols (PRISMA-P) statement (Additional file 1) and is registered with PROSPERO (https://www.crd.york.ac.uk/prospero/display_record.php?RecordID=85607) [22, 23]. We will track important protocol amendments on PROSPERO. We will use the PRISMA Extension Statement for Reporting of Systematic Reviews Incorporating Network Meta-Analyses of Health Care Interventions to guide reporting of this systematic review and NMA [24].

### Search strategy

We will customize literature search strategies to each database with the help of a research librarian, incorporating database-specific controlled vocabularies and text words. We will search MEDLINE (OVID interface, Epub Ahead of Print, In-Process & Other Non-Indexed Citations, 1946 to Present; Additional file 2), EMBASE (OVID interface, 1974 to Present), and the Cochrane Central Register of Controlled Trials (CENTRAL). We will not restrict searches by antibiotic type, date, publication status, or language to improve capture of varying antibiotic treatments and to minimize risk of publication bias as data from trials that compared now suboptimal or discontinued antibiotics may contribute to indirect evidence in a network. We will identify RCTs within MEDLINE and EMBASE using validated filters [25]. We will also search the World Health Organization’s International Clinical Trials Registry Platform and *meta*Register to identify ongoing trials. We will examine references of included RCTs and other pertinent publications to reduce the risk of failing to include a relevant RCT. We will rerun each of the database searches before submission of the final manuscript to capture any newly published RCTs.

### Eligibility criteria

We will include RCTs and quasi-RCTs (e.g., allocation is not truly random: by date of birth, day of the week, medical record number, or month of the year) of persons with suspected or confirmed SSTI treated with orally (e.g., in the form of tablets, liquids, or capsules) or parenterally (e.g., by injection subcutaneously, intramuscularly, or intravenously) administered antibiotic (mono- or combination) therapy that report results on at least one outcome of interest (see outcomes section below). Outcomes of interest are based on those recommended by the US Food and Drug Administration (FDA) and those commonly reported in RCTs of antibiotics for SSTI treatment [26]. Furthermore, eligible studies must contain at least one antibiotic intervention group (e.g., multi-arm and placebo-controlled trials are eligible). We will exclude studies of surgical site infections and in which an intervention consists of only topical antibiotic (e.g., in the form of powders, ointments, drops, or creams).

### Outcomes

Our primary outcome of interest is clinical success broadly defined as an improvement in signs and symptoms associated with SSTI at the test-of-cure (TOC) visit or closest clinical assessment to approximately 7–14 days after therapy completion. Secondary outcomes we are interested in are (1) early clinical success broadly defined as an improvement in signs and symptoms associated with SSTI at approximately 2–3 days after start of therapy, (2) mortality (all-cause or SSTI-associated) assessed approximately 28 days after therapy completion, (3) adverse events, (4) treatment duration, and (5) length of hospital stay.

### Study selection

We will pilot test a standardized form based on a priori inclusion and exclusion criteria and adapt it for each stage of screening: titles and abstracts, followed by full-text screening. Two independent reviewers will conduct screening after completion of calibration exercises. Disagreements will be resolved between the review pair, with the help of a third reviewer and correspondence with the potentially eligible study’s corresponding author when necessary. We will record reasons for exclusion of full texts and generate a PRIMSA flow diagram [23].

### Data extraction and management

We will pilot test and use a standardized Microsoft Excel form to extract data regarding study characteristics, participant demographics, antibiotic treatment, co-intervention(s) during treatment if any, analysis population for which a given outcome was ascertained (we will prefer extraction of outcome data from an intention-to-treat population over a clinically evaluable population reflecting success of empirical treatment, or we will extract relevant data from whatever population available), and outcomes of interest previously described. Reviewers will complete data extraction independently in pairs after performing calibration exercises. Disagreements will be resolved between the review pair and with the help of a third reviewer when necessary. We will contact corresponding authors of eligible RCTs for which data is missing or needs clarification. We will estimate mean and standard deviation (SD) for a continuous outcome from the median, range, and the size of the study sample when not available to be extracted [27].

### Risk of bias in individual randomized controlled trials

Independent reviewers will judge risk of bias in individual RCTs in duplicate according to the following domains outlined in the Cochrane risk of bias tool for RCTs: selection bias (random sequence generation and allocation concealment), performance bias (blinding of participants and personnel and other potential threats to validity), detection bias (blinding of outcome assessment and other potential threats to validity), attrition bias (incomplete outcome data), reporting bias (selective outcome reporting assessed by comparing outcomes reported in the protocol to those reported in the completed RCT whenever possible) and other sources of bias (e.g. for-profit funding). For a given domain, RCTs judged as definitely or probably being free of a given risk of bias will be considered low risk of bias, whereas RCTs judged as probably or definitely biased will be considered high risk of bias to reduce reporting of unclear bias assessments [28]. The review pair will resolve disagreements with the help of a third reviewer when necessary.

We will assess publication bias visually by judging asymmetry in the funnel plot and statistically provided at least 10 trials are included using the Egger test [29, 30].

### Direct comparison and network meta-analyses

We will synthesize direct evidence using traditional head-to-head meta-analyses when possible. Given the variety of antibiotic therapies available, we anticipate that not all will have been studied in head-to-head trials. Insufficient or total lack of direct evidence for possible antibiotic comparisons will warrant inclusion of indirect evidence to better approximate the value of an antibiotic regimen. Indirect evidence may also complement direct evidence when available and improve precision of an observed summary effect estimate. Therefore, direct, indirect, and a combination of direct and indirect evidence will inform each summary effect estimate reported whenever possible [31, 32]. Summary effect estimates will include odds ratio (OR) with corresponding 95% credible intervals (CrIs) for dichotomous outcomes and mean differences (MDs) with corresponding SDs for continuous outcomes. We will employ a random effects model for direct comparison meta-analyses and network meta-analyses within the Bayesian framework to account for expected within-study and between-study variability [32–35]. We will conduct all analyses in R software for statistical computing [36].

We will conduct NMAs provided the following assumptions are met. The transitivity (similarity) assumption gauges the appropriateness of combining evidence from individual trials of different treatments with a common comparator to enable analysis of indirect evidence. We will judge transitivity by comparing important clinical and methodological characteristics. The distribution of potential effect modifiers and the common comparator (e.g., dosage of antibiotic) should be similar between trials; otherwise, in the presence of large dissimilarity, NMA may be invalid [32, 37]. The consistency (coherence) assumption gauges the appropriateness of combining direct and indirect evidence for a given treatment comparison and is the result of intransitivity. We will assess consistency conceptually for treatment comparisons with direct and indirect evidence (a loop within a network) by comparing the size of the summary effect estimates and overlap between corresponding 95% CrIs of the two types of evidence. We will assess consistency statistically by applying the design-by-treatment-interaction model, which expands on the loop-specific approach not only to test loop but also design inconsistency (e.g., multi-arm trials) in the network. If summary effect estimates significantly vary in magnitude, CrIs do not overlap or there is a significant statistical inconsistency present, then we will seek loop-specific/local sources of heterogeneity [32, 37–39]. Further concerns that the transitivity and consistency assumptions have not been met may be remedied by adjusting for baseline characteristics in network meta-regression or splitting the network into subgroups, respectively [32]. This may improve our understanding of the relationship between different effect modifiers with antibiotic treatments and identify evidence in which we can have more confidence.

We will base NMAs on non-informative priors, for which the reference group may be the antibiotic most frequently trialed or placebo if there is wide spread in antibiotics trialed to facilitate interpretation. We will inspect model convergence using the Markov Chain Monte-Carlo simulation technique. The analysis will be run in three parallel chains, allowing for 20,000 burn-in simulations, followed by 100,000 simulations, which will be used to generate the outputs. We will also assess model convergence with the Gelman-Rubin diagnostic test and examination of Monte-Carlo errors [39–43].

### Assessment of heterogeneity

We will base investigation of methodological and clinical heterogeneity on a priori subgroups when appropriate. We still stratify SSTIs into the following groups based on severity: (1) uncomplicated SSTI (uSSTI) loosely defined as the presence of a skin lesion with local inflammation (erythema, edema, tenderness, and/or warmth) and (2) complicated SSTI (cSSTI) loosely defined as the presence of a skin lesion with local inflammation complicated by at least one of the following host-, anatomic site-, or organism-specific factors. Host-specific factors may include the need for surgical intervention and hospitalization, systemic symptoms (fever, hypotension, tachycardia, or altered mental status), comorbidities (diabetes, chronic liver or renal disease, asplenia, necrotizing peripheral neuropathy, vascular insufficiency, or immunocompromising disease), or any of the following signs or symptoms: bullae, hemorrhage, out-of-proportion pain, rapid progression, crepitus, and anesthesia. Anatomic site-specific factors may include depth (involvement of deeper skin structures: fascia or muscle), location (significant head or hand involvement), and size (> 9% of body surface area or > 75 cm^2^) of infection. Organism-specific factors may include antibiotic resistance of SSTI-causing bacteria (suspected or confirmed MRSA or other drug-resistant organisms) [2, 6, 44–46]. Additional subgroup analyses, stratified by severity when possible, may include age (children 0–17 years old, adults 18 to 64 years old, elderly persons 65 years old or older) and infection type. Impetigo, erysipelas, and cellulitis are infection types that do not involve deeper skin structures (infection of the epidermis, dermis, and subcutaneous fat, respectively) and are generally considered uSSTIs, unless complicated by other factors [6, 46]. We expect increased variability of infection types among cSSTIs and will consider those affecting deeper skin structures (necrotizing fasciitis, myositis, and gangrene) and persons with comorbidity (diabetic foot infection) specifically. Additional sources of heterogeneity among cSSTIs may be explained by subgroup analysis of infection in persons with comorbidities (diabetes, chronic liver or renal disease, asplenia, necrotizing peripheral neuropathy, vascular insufficiency, and immunocompromising disease) and with suspected or confirmed MRSA-associated infection.

We will set the criterion for statistical significance for the Chi^2^ test for heterogeneity at alpha = 0.10. Furthermore, an *I*^2^ value of > 50% will indicate the presence of substantial heterogeneity [47]. We will base allocation to a given subgroup on within-study definitions. We will subject studies of participants not easily categorized to subgroup analyses so long as it can be inferred that at least 80% of the population of interest meets subgroup criteria. Data permitting, we will perform meta-regression to further explore any important heterogeneity.

We expect some included RCTs to differ in their intervention and outcome definitions, especially regarding antibiotic dosing, breadth of adverse events captured, and timing of clinical assessments. We will be pragmatic in including these trials, but will perform sensitivity analyses to test the robustness of our results when trials whose differences are concerning are excluded. We will compare summary effect estimates from network comparisons of individual antibiotics and of antibiotic classes since we expect increased power to test differences in outcomes in network comparisons of antibiotic classes. We will conduct additional sensitivity analyses to test the robustness of our results to RCTs at high risk of bias.

### Network geometry and presentation of results

We will provide a narrative description of the connectedness of the antibiotic network, along with a network plot to visually assess its features, such as diversity of antibiotic treatments, frequency of antibiotic comparisons, and overall availability of evidence. The size of the nodes and width of the edges will be proportional to the amount of information they bear. The larger nodes and wider edges will contribute more to the NMA [32]. We will determine network nodes following data extraction, but will include individual antibiotics and antibiotic classes, to improve the robustness of the network. The extent of our confidence in the results will in part depend on the number of trials, participants, and events within each antibiotic comparison. Furthermore, the presence of both direct and indirect evidence for a given treatment comparison may increase our confidence and an unbalanced network (wide credible intervals around summary effect estimates) may reduce our confidence [31, 32, 37].

We will present network effect estimates and corresponding 95% CrIs in league tables and/or in forest plots against a standardized comparator. We will present the probabilities that a given treatment is the first, second, third, etcetera, best among all treatments for each outcome with cumulative probability rankograms [48, 49]. We will also generate surface under the cumulative ranking (SUCRA) curves to present the hierarchy of antibiotics. It may be inferred that antibiotics with larger SUCRA values, which can range from 0 to 100%, are better; however, a good rank or high SUCRA value may not correspond with a clinically important effect size. Therefore, it is important to consider the magnitude of effect sizes, certainty around the SUCRA values, and quality of the evidence informing the effect estimate of a given antibiotic comparison to gauge the value of an antibiotic [48, 49]. We may produce a rank-heat plot to present the treatment hierarchy for each outcome simultaneously [50].

### Certainty in effect estimates

We will assess certainty in NMA effect estimates in duplicate following calibration exercises for direct and indirect evidence using the Grading of Recommendations Assessment, Development and Evaluation (GRADE) approach [51]. We will judge certainty in direct evidence as very low, low, moderate, or high according to the following criteria: risk of bias, inconsistency, indirectness, imprecision, and publication bias. We will judge certainty in indirect evidence according to the same criteria with additional consideration for intransitivity. We will base judgments on evidence from first order loop comparisons (two head-to-head comparisons with a common comparator that contribute to a given indirect estimate) because their contribution to the indirect evidence pool is generally the greatest and most precise. The lower rating of the two direct estimates will be used as the rating of indirect evidence and may be rated down if we believe the transitivity assumption has not been met. If only indirect evidence is available for a treatment comparison, assessment of incoherence is precluded and intransitivity should more carefully be assessed. In the presence of both direct and indirect comparisons, we will apply the higher rating between the two types of evidence as the NMA effect estimate rating. In the presence of significant incoherence between direct and indirect evidence for a given comparison, we may rate the judgment down [51]. We will explicitly state the reasons for judgments and rating down for each outcome in the footnotes of evidence profiles or summary of finding tables generated in GRADEpro [52]. The value of an antibiotic should be dictated by the NMA and certainty in evidence (e.g., we might value an antibiotic with a lower ranking, but higher quality of evidence more compared to an antibiotic with a higher ranking, but very low quality of evidence) [51].

## Discussion

The high prevalence of SSTIs in community and hospital settings imposes a substantial socio-economic burden, which is exacerbated by their inappropriate clinical management [3, 4, 14, 15]. This, along with the lack of high-quality studies evaluating the relative effectiveness of the wide range of antibiotics used in the treatment of SSTIs, warrants the completion of a methodologically sound systematic review and, if possible, network meta-analysis to help better inform SSTI treatment recommendations.

Previous NMAs have had several weaknesses, which limit our confidence in their results and their implications for the clinical management of SSTIs. They were restrictive in their searches (by antibiotics and infection types), had limited generalizability, and were poorly conducted (e.g., screening and data extraction was not completed in duplicate, did not explore potential sources of heterogeneity and bias, and did not assess the overall quality of evidence) [18–21]. The proposed NMA will have several methodological strengths: (1) inclusion of a wider range of antibiotics and infection types; (2) a comprehensive search strategy; (3) screening and data extraction will be completed in duplicate by independent reviewers; (4) inclusion of a wider range of outcomes (e.g., duration of antibiotic treatment and hospital stay); (5) exploration of heterogeneity based on a priori subgroups and meta-regression, if possible; (6) application of GRADE to assess our certainty in the evidence; and (7) ease of interpretability of the comparative effectiveness of antibiotics (e.g., through presentation of results in league tables, cumulative probability rankograms, SUCRA curves, and/or a rank-heat plot).

Limitations to the proposed NMA may include variability in included RCT methods (e.g., heterogeneity in the definitions and time points used to assess outcomes of interest), considerable unexplained heterogeneity, uncertainty in effect estimates (e.g., less precision when only direct or indirect evidence is available), and unbalanced or underpowered networks. This review hopes to mediate some of these potential challenges through its comprehensiveness, a priori specified exploration of heterogeneity and sensitivity analyses, whenever possible.

With the wide range of antibiotics available for the treatment of SSTIs, we hope findings from this study will identify the antibiotic(s) with the most value in terms of patient-important outcomes to better inform health care professionals and policy makers resulting in improved evidence-based clinical management. Currently understudied antibiotic comparisons may be identified by evaluation of the network to guide future research and RCTs.

## Additional files

Additional file 1: Preferred Reporting Items for Systematic Review and Meta-Analysis Protocols (PRISMA-P) Checklist. (DOCX 32 kb)

Additional file 2: Medline Search Strategy. (DOC 37 kb)

## Abbreviations

CrI: Credible interval
cSSTI: Complicated skin and soft tissue infection
FDA: Food and Drug Administration
GRADE: Grading of Recommendations Assessment, Development and Evaluation
IDSA: Infectious Disease Society of America
MD: Mean difference
MRSA: Methicillin-resistant *Staphylococcus aureus*
NMA: Network meta-analysis
OR: Odds ratio
PRISMA-P: Preferred Reporting Items for Systematic Review and Meta-Analysis Protocols
RCT: Randomized controlled trial
SD: Standard deviation
SSTI: Skin and soft tissue infection
SUCRA: Surface under the cumulative ranking
TOC: Test-of-cure
US: United States
uSSTI: Uncomplicated skin and soft tissue infection
