# Supplementary material for: Antibiotic therapy for skin and soft tissue infections: a protocol for a systematic review and network meta-analysis
Source: Syst Rev. 2018 Sep 11;7:138. doi: 10.1186/s13643-018-0804-8 (PMC6134765; doi:10.1186/s13643-018-0804-8)
Supplement: Supplementary file 2 — Medline Search Strategy. (DOC 37 kb) [file 13643_2018_804_MOESM2_ESM.doc]

Database: OVID Medline Epub Ahead of Print, In-Process & Other Non-Indexed Citations, Ovid MEDLINE(R) Daily and Ovid MEDLINE(R) 1946 to Present

Search Strategy:

--------------------------------------------------------------------------------

1 (skin and soft tissue infection*).mp. [mp=title, abstract, original title, name of substance word, subject heading word, keyword heading word, protocol supplementary concept word, rare disease supplementary concept word, unique identifier, synonyms] (3973)

2 soft tissue infection*.mp. or exp Soft Tissue Infections/ (7438)

3 exp Skin Diseases, Bacterial/ or exp Methicillin-Resistant Staphylococcus aureus/ or exp Skin Diseases, Infectious/ or exp Staphylococcal Skin Infections/ or skin infection*.mp. or exp Skin/ or exp Staphylococcal Infections/ or streptococcal infection*.mp. or staphylococcal infection*.mp. or MRSA.mp. or skin.ti,ab. or exp dermis/ or exp epidermis/ (1387411)

4 (SSTI* or ABSSSI* or cSSTI*).mp. [mp=title, abstract, original title, name of substance word, subject heading word, keyword heading word, protocol supplementary concept word, rare disease supplementary concept word, unique identifier, synonyms] (1303)

5 impetigo.mp. or Exanthema/ or exp Impetigo/ (7997)

6 Orbital Cellulitis/ or exp Cellulitis/ or cellulitis.mp. (11726)

7 wound infection*.mp. or Wound Infection/ or (wound* adj2 infection*).ti,ab. (57770)

8 Diabetic Foot/ or Foot Diseases/ or Diabetes Complications/ or diabetic foot infection*.mp. or diabetic foot*.mp. (59235)

9 exp Wound Healing/ or exp Varicose Ulcer/ or venous leg ulcer*.mp. or exp Venous Insufficiency/ or exp Leg Ulcer/ or leg ulcer*.mp. (135967)

10 exp Erysipelas/ or erysipelas.mp. (2401)

11 exp Abscess/ or abscess.mp. or (skin adj4 abscess*).ti,ab. (77114)

12 exp Furunculosis/ or furunculosis.mp. (1798)

13 exp Carbuncle/ or carbunculosis.mp. (401)

14 exp Fasciitis, Necrotizing/ or fasciitis.mp. or exp Fasciitis/ (8052)

15 gangrene.mp. or exp Gangrene/ or exp Gas Gangrene/ (15008)

16 exp Anti-Bacterial Agents/ (644419)

17 (antibacter* or anti bacter* or antibiotic*).mp. (512364)

18 exp Tetracycline/ or tetracycline.mp. (38825)

19 lincosamide*.mp. or exp Lincosamides/ (8330)

20 macrolide*.mp. or exp Macrolides/ (106214)

21 aminoglycosides.mp. or exp Aminoglycosides/ (149537)

22 oxazolidinones.mp. or exp Oxazolidinones/ (8212)

23 glycopeptides.mp. or exp Glycopeptides/ (57556)

24 beta lactam.mp. or exp beta-Lactams/ or beta-lactam*.mp. or beta lactam*.mp. [mp=title, abstract, original title, name of substance word, subject heading word, keyword heading word, protocol supplementary concept word, rare disease supplementary concept word, unique identifier, synonyms] (144235)

25 penicillin*.mp. or exp Penicillins/ (103717)

26 carbapenem*.mp. or exp Carbapenems/ (15925)

27 cephalosporin*.mp. or exp Cephalosporins/ (49875)

28 monobactam*.mp. or exp Monobactams/ (1928)

29 antifolate*.mp. or exp Folic Acid Antagonists/ (55736)

30 exp Fluoroquinolones/ or fluroquinolones.mp. (29545)

31 (Azithromycin or Clarithromycin or Dirithromycin or Erythromycinor Flurithromycin or Josamycin or Midecamycin or Miocamycin).mp. (16717)

32 (Oleandomycin or Rokitamycin or Roxithromycin or Spiramycin or Troleandomycin or Tylosin or Ketolides or Telithromycin).mp. (7559)

33 (Cethromycin or Solithromycin).mp. (209)

34 (Clindamycin or Lincomycin or Pirlimycin).mp. (13696)

35 (Doxycycline or Chlortetracycline or Clomocycline or Demeclocycline or Lymecycline or Meclocycline or Metacycline).mp. (21940)

36 (Minocycline or Oxytetracycline or Penimepicycline or Rolitetracycline).mp. (16088)

37 (Streptomycin or Dihydrostreptomycin or Neomycin or Framycetin or Paromomycin or Ribostamycin or Kanamycin).mp. (52682)

38 (Amikacin or Arbekacin or Bekanamycin or Dibekacin or Tobramycin or Spectinomycin or Hygromycin B or Paromomycin).mp. (20745)

39 (Gentamicin# or Netilmicin or Sisomicin or Isepamicin or Verdamicin or Astromicin).mp. (19219)

40 (Eperezolid or Linezolid or Posizolid or Radezolid or Ranbezolid or Sutezolid or Tedizolid).mp. (5194)

41 (Vancomycin or Oritavancin or Telavancin or Teicoplanin or Dalbavancin or Ramoplanin).mp. (28240)

42 (Amoxicillin or Ampicillin or Pivampicillin or Hetacillin or Bacampicillin or Metampicillin or Talampicillin).mp. (42504)

43 (Epicillin or Carbenicillin or Carindacillin or Ticarcillin or Temocillin or ureidopenicillins or Azlocillin).mp. (5852)

44 (Benzathine benzylpenicillin or Procaine benzylpenicillin or Azidocillin or Penamecillin or Phenoxymethylpenicillin).mp. (604)

45 (Propicillin or Pheneticillin or Cloxacillin or Dicloxacillin or Flucloxacillin or Oxacillin or Meticillin).mp. (9946)

46 (Nafcillin or Carbapenems or Biapenem or Ertapenem or Doripenem or Imipenem or Meropenem).mp. (19547)

47 (Panipenem or Cephalosporins or Cefazolin or Cefacetrile or Cefadroxil or Cefalexin or Cefaloglycin).mp. (30371)

48 (Cefalonium or Cefaloridine or Cefalotin or Cefapirin or Cefatrizine or Cefazedone or Cefazaflur).mp. (414)

49 (Cefradine or Cefroxadine or Ceftezole or Cephalosporins or Cefaclor or Cefamandole or Cefminox).mp. (28103)

50 (Cefonicid or Ceforanide or Cefotiam or Cefprozil or Cefbuperazone or Cefuroxime or Cefuzonam or cephamycin).mp. (6329)

51 (Cefoxitin or Cefotetan or Cefmetazole or carbacephem or Loracarbef or Cefixime or Ceftriaxone or Ceftazidime).mp. (24132)

52 (Cefoperazone or Cefcapene or Cefdaloxime or Cefdinir or Cefditoren or Cefetamet or Cefmenoxime or Cefodizime).mp. (4094)

53 (Cefotaxime or Cefpimizole or Cefpiramide or Cefpodoxime or Cefsulodin or Cefteram or Ceftibuten or Ceftiolene).mp. (11622)

54 (Ceftizoxime or oxacephem or Flomoxef or Latamoxef or Cefepime or Cefozopran or Cefpirome or Cefquinome).mp. (5887)

55 (Ceftobiprole or Ceftaroline fosamil or Ceftolozane or Monobactams or Aztreonam or Tigemonam or Carumonam).mp. (4106)

56 (Nocardicin A Beta-lactam or Beta-lactamase inhibitor or clavulanic acid or Imipenem or cilastatin or Ampicillin flucloxacillin).mp. (15743)

57 (Ampicillin sulbactam or Sultamicillin or Ceftazidime avibactam or Piperacillin tazobactam or Antifolates).mp. (5691)

58 (Sulfaisodimidine or Sulfamethizole or Sulfadimidine or Sulfapyridine or Sulfafurazole or Sulfanilamide or Prontosil).mp. (8510)

59 (Sulfathiazole or Sulfathiourea or Sulfamethoxazole or Sulfadiazine or Sulfamoxole or Sulfadimethoxine or Sulfadoxine).mp. (25505)

60 (Sulfalene or Sulfametomidine or Sulfametoxydiazine or Sulfamethoxypyridazine or Sulfaperin or Sulfamerazine).mp. (1517)

61 (Sulfaphenazole or Sulfamazone or Sulfacetamide or Sulfadicramide or Sulfametrole or Trimethoprim sulfamethoxazole or Septra).mp. (12246)

62 (Fluoroquinolones Quinolones or Cinoxacin or Flumequine or Nalidixic acid or Oxolinic acid or Pipemidic acid).mp. (7361)

63 (Piromidic acid or Rosoxacin or Ciprofloxacin or Enoxacin or Fleroxacin or Lomefloxacin or Nadifloxacin or Ofloxacin).mp. (32254)

64 (Norfloxacin or Pefloxacin or Rufloxacin or Balofloxacin or Grepafloxacin or Levofloxacin or Pazufloxacin).mp. (12202)

65 (Sparfloxacin or Temafloxacin or Tosufloxacin or Besifloxacin or Clinafloxacin or Garenoxacin).mp. (2147)

66 (Gemifloxacin or Moxifloxacin or Gatifloxacin or Sitafloxacin or Trovafloxacin Alatrofloxacin).mp. (5667)

67 (Prulifloxacin or Anaerobic DNA inhibitor: or Metronidazole or Tinidazole or Ornidazole or Nitrofurantoin).mp. (23393)

68 (Furazolidone or Nifurtoinol or RNA synthesis inhibitor* or Rifampicin or Rifabutin or Rifapentine or Rifaximin).mp. (19892)

69 daptomycin.mp. or exp Daptomycin/ (2743)

70 linezolid.mp. or exp Linezolid/ (5092)

71 tigecycline.mp. (2648)

72 clavulanate.mp. (4732)

73 cephalexin.mp. or exp Cephalexin/ (4562)

74 moxalactam.mp. or exp Moxalactam/ (1505)

75 cilastin.mp. (36)

76 pristinamycin.mp. or exp Pristinamycin/ (484)

77 minomycin.mp. or exp Minocycline/ (5374)

78 fosfomycin.mp. or exp Fosfomycin/ (2825)

79 cotrimoxazole.mp. or exp Trimethoprim, Sulfamethoxazole Drug Combination/ (8512)

80 randomized controlled trial.pt. (453665)

81 random allocation/ (93266)

82 double-blind method/ (144118)

83 single-blind method/ (24623)

84 randomi?ed controlled trial$.mp. (616489)

85 Randomi?ed clinical trial$.mp. (41642)

86 controlled clinical trial.pt. (92160)

87 ((singl$ or double$ or trebl$ or tripl$) adj25 (blind$ or mask$)).mp. (218415)

88 random$.mp. (1172639)

89 cross-over studies.sh. (42047)

90 placebo.mp. (192581)

91 animals/ not humans/ (4393253)

92 1 or 2 or 3 or 4 or 5 or 6 or 7 or 8 or 9 or 10 or 11 or 12 or 13 or 14 or 15 (1632948)

93 16 or 17 or 18 or 19 or 20 or 21 or 22 or 23 or 24 or 25 or 26 or 27 or 28 or 29 or 30 or 31 or 32 or 33 or 34 or 35 or 36 or 37 or 38 or 39 or 40 or 41 or 42 or 43 or 44 or 45 or 46 or 47 or 48 or 49 or 50 or 51 or 52 or 53 or 54 or 55 or 56 or 57 or 58 or 59 or 60 or 61 or 62 or 63 or 64 or 65 or 66 or 67 or 68 or 69 or 70 or 71 or 72 or 73 or 74 or 75 or 76 or 77 or 78 or 79 (1057002)

94 80 or 81 or 82 or 83 or 84 or 85 or 86 or 87 or 88 or 89 or 90 (1317894)

95 92 and 93 and 94 (13410)

96 95 not 91 (12405)

***************************
